# The genome sequence of the lesser treble-bar moth, *Aplocera efformata* (Guenée, 1857)

**DOI:** 10.12688/wellcomeopenres.18595.1

**Published:** 2022-12-15

**Authors:** Douglas Boyes, University of Oxford and Wytham Woods Genome Acquisition Lab, Darwin Tree of Life Barcoding collective, Wellcome Sanger Institute Tree of Life programme, Wellcome Sanger Institute Scientific Operations: DNA Pipelines collective, Tree of Life Core Informatics collective, Marc Palmada-Flores, Darwin Tree of Life Consortium

**Affiliations:** 1 UK Centre for Ecology and Hydrology, Wallingford, Oxfordshire, UK; 2 Department of Medicine and Life Sciences (MELIS), Institut de Biologia Evolutiva, Universitat Pompeu Fabra-CSIC, Barcelona, Spain, 08003, Spain

**Keywords:** Aplocera efformata, lesser treble-bar, genome sequence, chromosomal, Lepidoptera

## Abstract

We present a genome assembly from an individual female *Aplocera efformata* (the lesser treble-bar; Arthropoda; Insecta; Lepidoptera; Geometridae). The genome sequence is 350 megabases in span. Most of the assembly (99.97%) is scaffolded into 32 chromosomal pseudomolecules, with W and Z sex chromosomes assembled. The complete mitochondrial genome was also assembled and is 15.4 kilobases in length. Gene annotation of this assembly on Ensembl has identified 11,393 protein coding genes.

## Species taxonomy

Eukaryota; Metazoa; Ecdysozoa; Arthropoda; Hexapoda; Insecta; Pterygota; Neoptera; Endopterygota; Lepidoptera; Glossata; Ditrysia; Geometroidea; Geometridae; Larentiinae; *Aplocera*; *Aplocera efformata* (Guenée, 1857) (NCBI:txid934917).

## Background

The lesser treble-bar, *Aplocera efformata* (Guenée, 1857), is a geometer moth within the subfamily Larentiinae (family Geometridae) composed of carpets, pugs and allies ( Waring & Townsend, 2017). It is hard to distinguish from its sister species, the treble-bar ( *Aplocera plagiata*), as both are grey with three dark cross-bands in their pointed forewings. However, the lesser treble-bar species is slightly smaller, with a forewing length of 16–19 mm, and displays less intense dark cross-bands and lighter forewings. Its abdomen also has a shorter taper to the apex compared to the very pointed abdomen of the treble-bar ( Townsend *et al.*, 2010; Waring & Townsend, 2017).

The lesser treble-bar’s range extends from Morocco across southern and central Europe, reaching Anatolia to the east and southern Scandinavia to the north ( Bálint *et al.*, 2016).

The preferred habitat of *A. efformata* is hot, dry grasslands, mainly on sandy or calcareous ground, though it is sometimes encountered in regions such as sea-cliffs, woodland rides, abandoned quarries, field margins and gardens. *A. efformata* presents two generations of flight seasons, which are easily disturbed by day, overwinters as larvae and pupates underground ( Bálint *et al.*, 2016; Waring & Townsend, 2017). 

In Europe, the species has been suffering a decline in population, being threatened by the diminution of their favoured habitat ( Bálint *et al.*, 2016). We predict that the Darwin Tree of Life assembly presented here will be an important tool for further examination of its population dynamics.

## Genome sequence report

**Figure 1.**
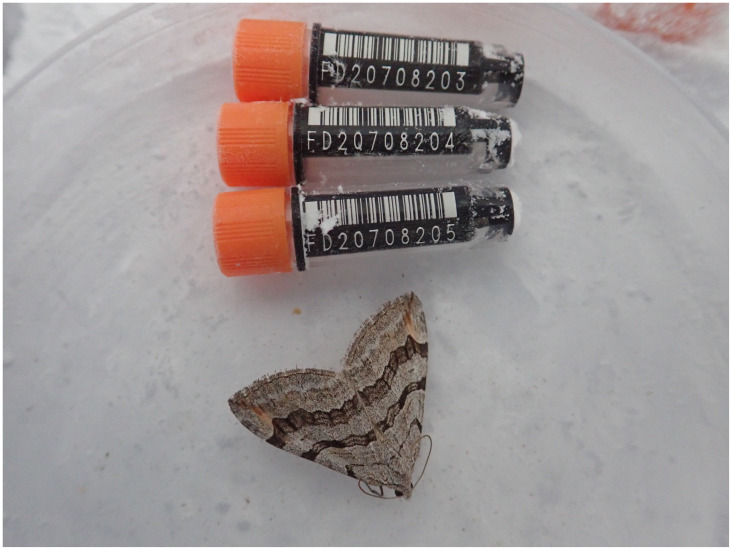
Image of the female *Aplocera efformata* specimen from which the genome was sequenced. The ilAplEffo1 specimen was used to generate Pacific Biosciences, 10X genomics, Hi-C and RNA-Seq data.

The genome was sequenced from a single female *A. efformata* ( Figure 1) collected from Wytham Woods, Berkshire, UK (latitude 51.772, longitude –1.338). A total of 53-fold coverage in Pacific Biosciences single-molecule circular consensus (HiFi) long reads and 128-fold coverage in 10X Genomics read clouds were generated. Primary assembly contigs were scaffolded with chromosome conformation Hi-C data. Manual assembly curation corrected four misjoins which reduced the scaffold number by 7.27%.

**Figure 2.**
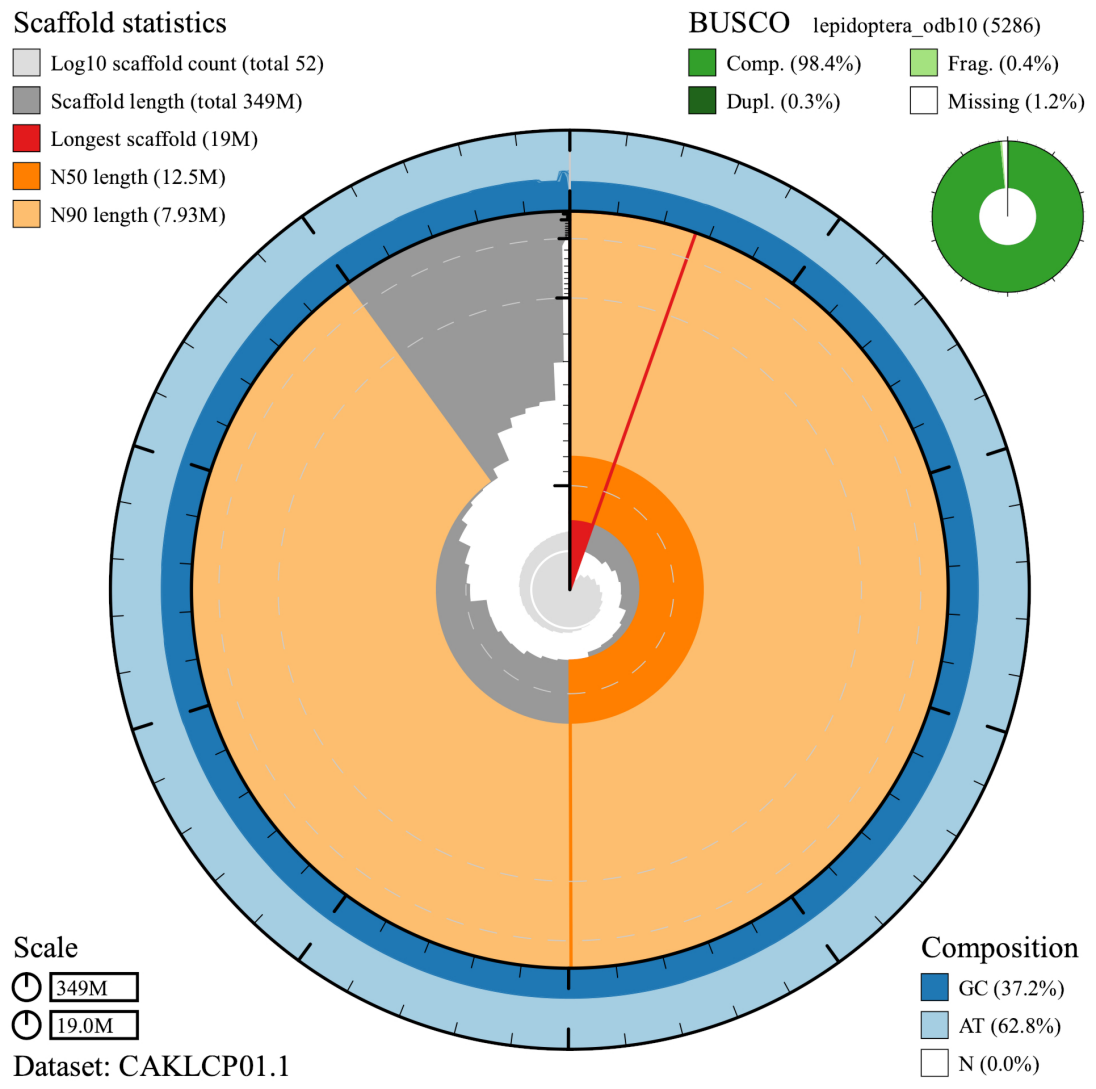
Genome assembly of *Aplocera efformata*, ilAplEffo1.1: metrics. The main plot is divided into 1,000 size-ordered bins around the circumference with each bin representing 0.1% of the 349,498,550 bp assembly. The distribution of chromosome lengths is shown in dark grey with the plot radius scaled to the longest chromosome present in the assembly (19,009,616 bp, shown in red). Orange and pale-orange arcs show the N50 and N90 chromosome lengths (12,527,553 and 7,930,759 bp), respectively. The pale grey spiral shows the cumulative chromosome count on a log scale with white scale lines showing successive orders of magnitude. The blue and pale-blue area around the outside of the plot shows the distribution of GC, AT and N percentages in the same bins as the inner plot. A summary of complete, fragmented, duplicated and missing BUSCO genes in the lepidoptera_odb10 set is shown in the top right. An interactive version of this figure is available at https://blobtoolkit.genomehubs.org/view/ilAplEffo1.1/dataset/CAKLCP01.1/snail.

The final assembly has a total length of 349 Mb in 51 sequence scaffolds with a scaffold N50 of 12.5 Mb ( Table 1). Most of the assembly sequence (99.97%) was assigned to 32 chromosomal-level scaffolds, representing 30 autosomes (numbered by sequence length) and the W and Z sex chromosomes ( Figure 2– Figure 5; Table 2).

**Table 1.** Genome data for *A. efformata*, ilAplEffo1.1.

*Project accession data*	
Assembly identifier	ilAplEffo1.1
Species	*Aplocera efformata*
Specimen	ilAplEffo1 (genome assembly, Hi-C, RNA-Seq)
NCBI taxonomy ID	934917
BioProject	PRJEB47323
BioSample ID	SAMEA8603170
Isolate information	Female, thorax tissue (genome assembly), head tissue (Hi-C), abdomen tissue (RNA-Seq)
*Raw data accessions*	
PacificBiosciences SEQUEL II	ERR6939267
10X Genomics Illumina	ERR6688760-ERR6688763
Hi-C Illumina	ERR6688759
PolyA RNA-Seq Illumina	ERR9435023
*Genome assembly*	
Assembly accession	GCA_921293045.1
*Accession of alternate haplotype*	GCA_921293035.1
Span (Mb)	350
Number of contigs	55
Contig N50 length (Mb)	12.5
Number of scaffolds	51
Scaffold N50 length (Mb)	12.5
Longest scaffold (Mb)	15.0
BUSCO * genome score	C:98.4%[S:98.1%,D:0.3%],F:0. 4%,M:1.2%,n:5,286
**Genome annotation**	
Number of protein-coding genes	11,393

*BUSCO scores based on the lepidoptera_odb10 BUSCO set using v5.3.2. C = complete [S = single copy, D = duplicated], F = fragmented, M = missing, n = number of orthologues in comparison. A full set of BUSCO scores is available at https://blobtoolkit.genomehubs.org/view/ilAplEffo1.1/dataset/CAKLCP01.1/busco.

**Figure 3.**
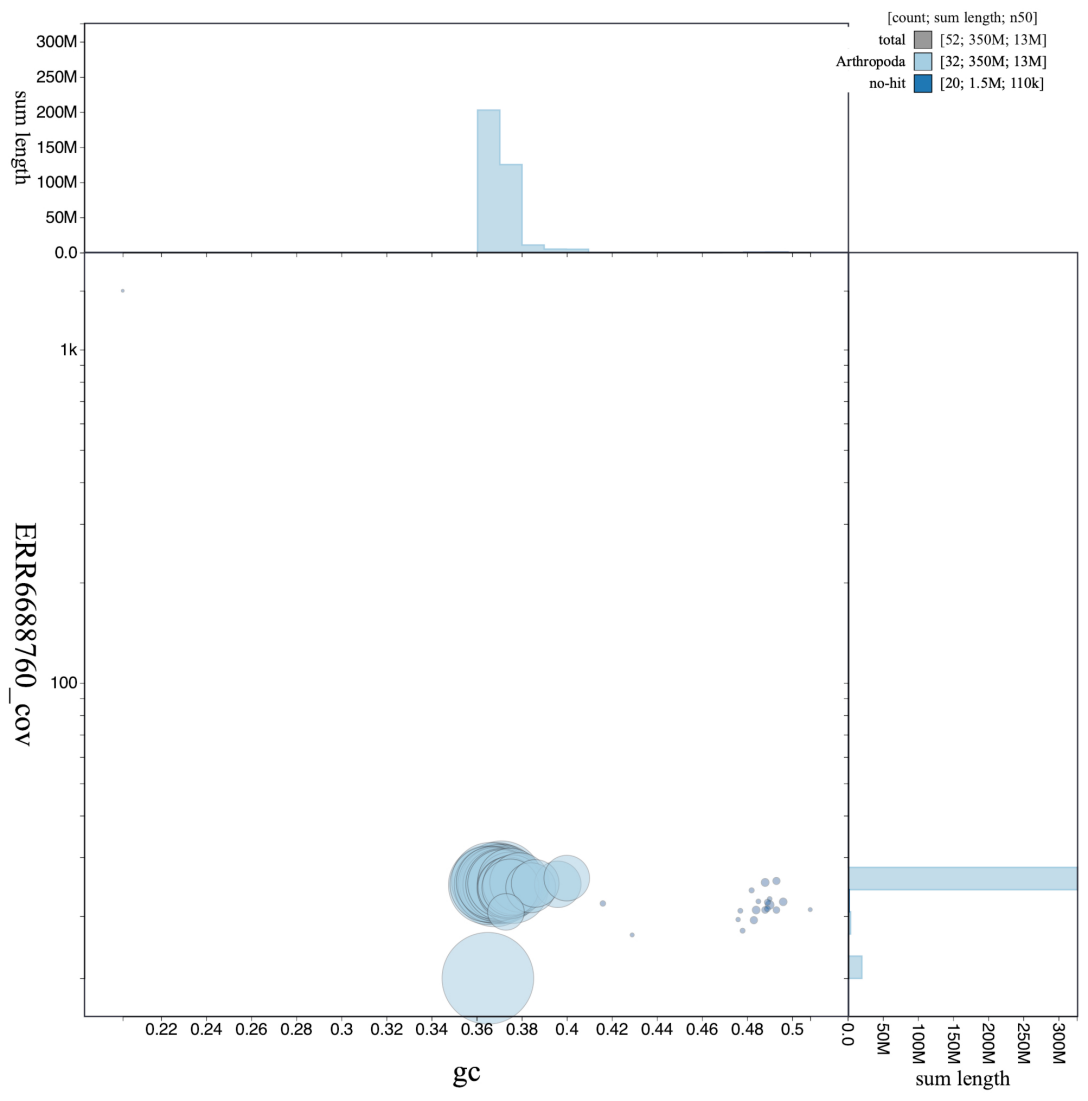
Genome assembly of *Aplocera efformata*, ilAplEffo1.1: GC coverage. BlobToolKit GC-coverage plot. Scaffolds are coloured by phylum. Circles are sized in proportion to scaffold length. Histograms show the distribution of scaffold length sum along each axis. An interactive version of this figure is available at https://blobtoolkit.genomehubs.org/view/ilAplEffo1.1/dataset/CAKLCP01.1/blob.

**Figure 4.**
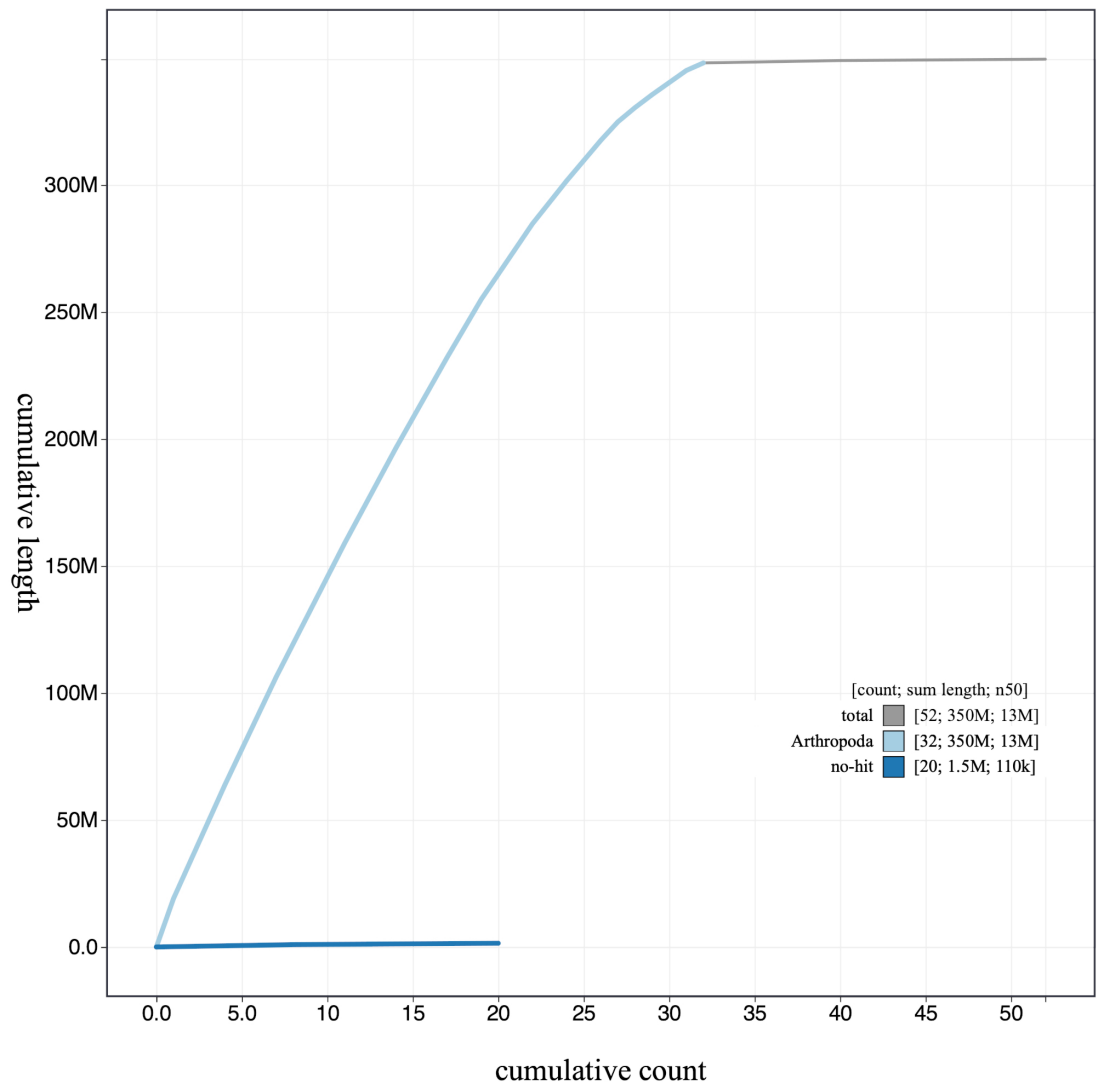
Genome assembly of *Aplocera efformata*, ilAplEffo1.1: cumulative sequence. BlobToolKit cumulative sequence plot. The grey line shows cumulative length for all scaffolds. Coloured lines show cumulative lengths of scaffolds assigned to each phylum using the buscogenes taxrule. An interactive version of this figure is available at https://blobtoolkit.genomehubs.org/view/ilAplEffo1.1/dataset/CAKLCP01.1/cumulative.

**Figure 5.**
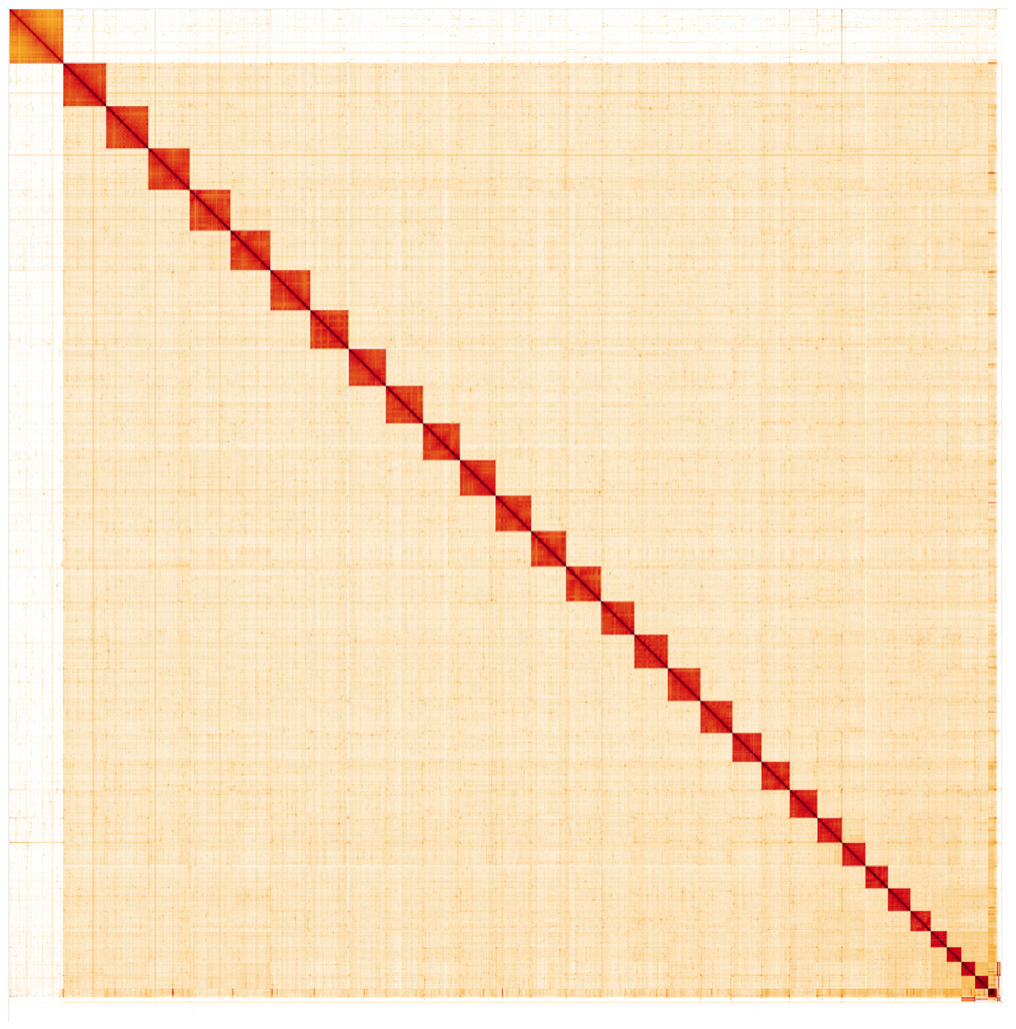
Genome assembly of *Aplocera efformata*, ilAplEffo1.1: Hi-C contact map. Hi-C contact map of the ilAplEffo1.1 assembly, visualised in HiGlass. Chromosomes are arranged in size order from left to right and top to bottom. The interactive Hi-C map can be viewed at https://genome-note-higlass.tol.sanger.ac.uk/l/?d=DFH-u6PYSz6oMane3MisUg.

**Table 2.** Chromosomal pseudomolecules in the genome assembly of *A. efformata*, ilAplEffo1.1.

INSDC accession	Chromosome	Size (Mb)	GC%
OV121039.1	1	15.04	37.1
OV121040.1	2	15.03	36.9
OV121041.1	3	14.63	36.8
OV121042.1	4	14.27	37.1
OV121043.1	5	14.13	37
OV121044.1	6	14.06	36.5
OV121045.1	7	13.39	36.6
OV121046.1	8	13.18	36.7
OV121047.1	9	13.08	36.7
OV121048.1	10	12.96	36.5
OV121049.1	11	12.55	37
OV121050.1	12	12.53	37
OV121051.1	13	12.41	36.9
OV121052.1	14	12.18	36.8
OV121053.1	15	11.81	37
OV121054.1	16	11.73	36.7
OV121055.1	17	11.5	37.1
OV121056.1	18	11.44	37.6
OV121057.1	19	10.22	37.1
OV121058.1	20	9.95	37.5
OV121059.1	21	9.61	37.5
OV121060.1	22	8.7	37.4
OV121061.1	23	8.21	37.4
OV121062.1	24	8	37.6
OV121063.1	25	7.93	37.9
OV121064.1	26	7.3	37.5
OV121065.1	27	4.84	39.6
OV121066.1	28	5.59	38.4
OV121067.1	29	5.09	38.6
OV121068.1	30	4.67	40
OV121069.1	W	2.98	37.3
OV121038.1	Z	19.01	36.5
OV121070.1	MT	0.02	20.4
-	Unplaced	1.45	48.4

The assembly has a BUSCO v5.3.2 ( Manni *et al.*, 2021) completeness of 98.4% (single 98.1%, duplicated 0.3%) using the lepidoptera_odb10 reference set ( *n* = 5,286). While not fully phased, the assembly deposited is of one haplotype. Contigs corresponding to the second haplotype have also been deposited.

## Genome annotation report

The GCA_921293045.1 genome was annotated using the Ensembl rapid annotation pipeline ( Table 1). The resulting annotation includes 19,297 transcribed mRNAs from 11,393 protein-coding and 1,074 non-coding genes.

## Methods

### Sample acquisition and nucleic acid extraction

A single female *A. efformata* specimen (ilAplEffo1) was collected in Wytham Woods, Berkshire, UK (latitude 51.772, longitude –1.338) by Douglas Boyes (University of Oxford), using a light trap. The sample was identified by Douglas Boyes and snap-frozen on dry ice.

DNA was extracted at the Tree of Life laboratory, Wellcome Sanger Institute. The ilAplEffo1 sample was weighed and dissected on dry ice with head tissue set aside for Hi-C sequencing. Thorax tissue was disrupted using a Nippi Powermasher fitted with a BioMasher pestle. Fragment size analysis of 0.01–0.5 ng of DNA was then performed using an Agilent FemtoPulse. High molecular weight (HMW) DNA was extracted using the Qiagen MagAttract HMW DNA extraction kit. Low molecular weight DNA was removed from a 200 ng aliquot of extracted DNA using 0.8X AMpure XP purification kit prior to 10X Chromium sequencing; a minimum of 50 ng DNA was submitted for 10X sequencing. HMW DNA was sheared into an average fragment size of 12–20 kb in a Megaruptor 3 system with speed setting 30. Sheared DNA was purified by solid-phase reversible immobilisation using AMPure PB beads with a 1.8X ratio of beads to sample to remove the shorter fragments and concentrate the DNA. The concentration of the sheared and purified DNA was assessed using a Nanodrop spectrophotometer and Qubit Fluorometer and Qubit dsDNA High Sensitivity Assay kit. Fragment size distribution was evaluated by running the sample on the FemtoPulse system.

RNA was extracted from the abdomen tissue of ilAplEffo1 in the Tree of Life Laboratory at the WSI using TRIzol, according to the manufacturer’s instructions. RNA was then eluted in 50 μl RNAse-free water and the RNA concentration was assessed using a Nanodrop spectrophotometer and Qubit Fluorometer using the Qubit RNA Broad-Range (BR) Assay kit. Analysis of the integrity of the RNA was done using Agilent RNA 6000 Pico Kit and Eukaryotic Total RNA assay.

### Sequencing

Pacific Biosciences HiFi circular consensus and 10X Genomics read cloud DNA sequencing libraries were constructed according to the manufacturers’ instructions. Poly(A) RNA-Seq libraries were constructed using the NEB Ultra II RNA Library Prep kit. DNA and RNA sequencing was performed by the Scientific Operations core at the WSI on Pacific Biosciences SEQUEL II (HiFi), Illumina NovaSeq 6000 and Illumina HiSeq 4000 (RNA-Seq) instruments. Hi-C data were also generated from the remaining head tissue of ilAplEffo1 using the Arima v2 Hi-C kit and sequenced on an Illumina NovaSeq 6000 instrument.

### Genome assembly

Assembly was carried out with Hifiasm ( Cheng *et al.*, 2021) and haplotypic duplication was identified and removed with purge_dups ( Guan *et al.*, 2020). One round of polishing was performed by aligning 10X Genomics read data to the assembly with longranger align, calling variants with freebayes ( Garrison & Marth, 2012). The assembly was then scaffolded with Hi-C data ( Rao *et al.*, 2014) using SALSA2 ( Ghurye *et al.*, 2019). The assembly was checked for contamination as described previously ( Howe *et al.*, 2021). Manual curation was performed using HiGlass ( Kerpedjiev *et al.*, 2018) and PretextView ( Harry, 2022). The mitochondrial genome was assembled using MitoHiFi ( Uliano-Silva *et al.*, 2021), which performs annotation using MitoFinder ( Allio *et al.*, 2020). The genome was analysed within the BlobToolKit environment ( Challis *et al.*, 2020), generating BUSCO scores. Table 3 contains a list of all software tool versions used, where appropriate.

**Table 3.** Software tools and versions used.

Software tool	Version	Source
BlobToolKit	3.3.2	( Challis *et al.*, 2020)
freebayes	1.3.1-17-gaa2ace8	( Garrison & Marth, 2012)
Hifiasm	0.15.3	( Cheng *et al.*, 2021)
HiGlass	1.11.6	Kerpedjiev *et al.*, 2018
longranger align	2.2.2	https://support.10xgenomics.com/genome-exome/ software/pipelines/latest/advanced/other-pipelines
MitoHiFi	2.0	( Uliano-Silva *et al.*, 2021)
PretextView	0.1.x	( Harry, 2022)
purge_dups	1.2.3	( Guan *et al.*, 2020)
SALSA2	2.2	( Ghurye *et al.*, 2019)

### Genome annotation

The Ensembl gene annotation system ( Aken *et al.*, 2016) was used to generate annotation for the *A. efformata* assembly (GCA_921293045.1). Annotation was created primarily through alignment of transcriptomic data to the genome, with gap filling via protein to-genome alignments of a select set of proteins from UniProt (( UniProt Consortium, 2019)).

## Data Availability

European Nucleotide Archive: *Aplocera efformata* (lesser treble-bar). Accession number PRJEB47323; https://identifiers.org/ena.embl/PRJEB47323 ( Wellcome Sanger Institute, 2022) The genome sequence is released openly for reuse. The *A. efformata* genome sequencing initiative is part of the Darwin Tree of Life (DToL) project. All raw sequence data and the assembly have been deposited in INSDC databases. The genome will be annotated using the RNA-Seq data and presented through the Ensembl pipeline at the European Bioinformatics Institute. Raw data and assembly accession identifiers are reported in Table 1.

## Author information

Members of the University of Oxford and Wytham Woods Genome Acquisition Lab are listed here: https://doi.org/10.5281/zenodo.4789928.

Members of the Darwin Tree of Life Barcoding collective are listed here: https://doi.org/10.5281/zenodo.4893703.

Members of the Wellcome Sanger Institute Tree of Life programme are listed here: https://doi.org/10.5281/zenodo.4783585.

Members of Wellcome Sanger Institute Scientific Operations: DNA Pipelines collective are listed here: https://doi.org/10.5281/zenodo.4790455.

Members of the Tree of Life Core Informatics collective are listed here: https://doi.org/10.5281/zenodo.5013541.

Members of the Darwin Tree of Life Consortium are listed here: https://doi.org/10.5281/zenodo.4783558.
