# Supplementary material for: The genome sequence of the lesser treble-bar moth, Aplocera efformata (Guenée, 1857)
Source: Wellcome Open Res. 2022 Dec 15;7:303. [Version 1] doi: 10.12688/wellcomeopenres.18595.1 (PMC10628359; doi:10.12688/wellcomeopenres.18595.1)
Supplement: Peer review history

# Reviewer report: Reviewer response for version 1

**DOI:** 10.21956/wellcomeopenres.20620.r69217

**Published:** 2023-11-08

**Authors:** Muzafar Riyaz

**Affiliations:** 1 Xavier Research Foundation, St Xavier's College, Palayamkottai, Tamil Nadu, India

The article titled "The genome sequence of the lesser treble-bar moth, Aplocera efformata (Guenée, 1857)" presents a comprehensive analysis of the genome of Aplocera efformata, shedding light on the genetic makeup of this lesser-known species within the Lepidoptera order. The study provides valuable insights into the evolutionary and ecological aspects of this geometer moth, making it a significant contribution to the field of genomics and entomology.

 The authors meticulously detail the methodology employed in genome sequencing, which involved a combination of advanced technologies such as Pacific Biosciences HiFi circular consensus and 10X Genomics read cloud DNA sequencing libraries. The assembly process, outlined in the article, is notable for its depth and precision, resulting in a high-quality genome assembly of A. efformata. The inclusion of Hi-C data in scaffolding the assembly ensures the chromosomal-level accuracy, which is crucial for understanding the genetic architecture and chromosomal organization of the species.

 The article provides a thorough overview of the lesser treble-bar moth, its taxonomy, habitat, and distinguishing features, setting the stage for the importance of genomic analysis in understanding its population dynamics and evolutionary history. The detailed genome annotation, involving alignment of transcriptomic data and protein-to-genome alignments, enhances our understanding of the genetic elements within this species, including protein-coding and non-coding genes.

 One of the key strengths of the article lies in its commitment to transparency and accessibility. The authors have made the genome sequence openly available, promoting further research and ensuring the data's usability by the scientific community. The comprehensive data availability section, including accession numbers and relevant links, enhances the reproducibility and credibility of the study. Additionally, the article effectively integrates visualizations, such as Hi-C contact maps and genome assembly metrics, enhancing the reader's understanding of the methodology and results. These visual aids contribute to the clarity of the presented information.

 The genome sequence of the lesser treble-bar moth, Aplocera efformata (Guenée, 1857)" stands out as a well-executed genomic study, providing valuable insights into the lesser treble-bar moth's genetic makeup. The meticulous methodology, detailed annotations, and transparent data sharing make this article a significant resource for researchers in genomics, evolutionary biology, and entomology.

Are sufficient details of methods and materials provided to allow replication by others?

Yes

Is the rationale for creating the dataset(s) clearly described?

Yes

Are the datasets clearly presented in a useable and accessible format?

Yes

Are the protocols appropriate and is the work technically sound?

Yes

Reviewer Expertise:

Genomics, Phylogenomics, Mitogenomics, and Evolutionary Biology

I confirm that I have read this submission and believe that I have an appropriate level of expertise to confirm that it is of an acceptable scientific standard.

# Reviewer report: Reviewer response for version 1

**DOI:** 10.21956/wellcomeopenres.20620.r69223

**Published:** 2023-11-06

**Authors:** Niklas Wahlberg

**Affiliations:** 1 Lund university, Lund, Sweden

The report on the genome of *Aplocera efformata* (Lepidoptera: Geometridae) follows the standard format for genome notes, of which more than 200 have been published already for butterflies and moths. This is an amazing resource for the scientific community! In this particular case, the genome of *A. efformata* will be interesting to compare to the closely related *A. plagiata*, from which it is difficult to distinguish (as mentioned in the article).

 The methods section has been written clearly, and it is good that the format has developed, with all the programs being listed along with the version that was used for assembling the genome. I have nothing more to add, it seems that Lepidoptera genomes are easy to sequence and easy to assemble.

Are sufficient details of methods and materials provided to allow replication by others?

Yes

Is the rationale for creating the dataset(s) clearly described?

Yes

Are the datasets clearly presented in a useable and accessible format?

Yes

Are the protocols appropriate and is the work technically sound?

Yes

Reviewer Expertise:

Phylogenomics, systematics, Lepidoptera, Geometridae

I confirm that I have read this submission and believe that I have an appropriate level of expertise to confirm that it is of an acceptable scientific standard.

# Reviewer report: Reviewer response for version 1

**DOI:** 10.21956/wellcomeopenres.20620.r55797

**Published:** 2023-03-28

**Authors:** Jaakko L.O. Pohjoismäki

**Affiliations:** 1 Department of Environmental and Biological Sciences, University of Eastern Finland, Joensuu, Finland

The genome release of the lesser treble-bar moth by Douglas Boyes and the DToL crew is another nice addition to the growing number of Lepidopteran reference genomes. Also, the presented species is not that trivial and could offer some interesting insights into taxonomy and biology of these moths. 

 The provided metrics seem in agreement with what is required from reference genomes, but my understanding of the bioinformatic work is not sufficient to evaluate the used approach further. Instead, I try to contribute some thoughts for the taxonomic and faunistic parts of the report.

**Background**

 You could point out that *A. efformata* is a monophagous species on *Hypericum*. This might be relevant, as monophagy vs polyphagy could be somehow reflected by the insect genomes (adaptations to plant secondary compounds etc). Perhaps something to address in the future? If you have some speculation to offer about this (adaptive signatures, specialized detoxifying enzymes etc), please feel free to add a couple of sentences at the end of the section.

 […] geometer moth […] -> geometrid moth

 […] decline in population […] The sentence reads odd. Please reformulate.

**Methods:**

 The trapping date is not provided.

 A representative of the heterogametic sex was analyzed, which is great as it is not trivial with night active moths (males more often caught on light). However, please assign a voucher specimen for the genome and provide details of its location (name of the collection, storing institute storing) for future reference.

 I would have chosen thorax for RNA as there are probably more tissue types present. Moreover, the female abdomen is often full of eggs (note that the specimen looks relatively young, wings and scales intact, so probably has not laid its eggs yet) that mainly have a limited set of maternal RNAs for the early embryonic development. Was this reflected by the RNA-seq data? If so, comment this in the genome annotation report.

 As pointed out in the background chapter, *A. efformata* is very similar to *plagiata*. While I trust the skill of your taxonomic expert, I nevertheless extracted the *Co1* sequence from the mitochondrial genome you provided and it seems to agree with *efformata*. The authors could point out that the species identity can be confirmed in this way using DNA-barcoding (95.5 % pairwise identity between the *Co1 *sequence from the two species, so a nice separation). It would also not harm to mention here the BINs for the different *Aplocera* species in BOLD for comparison.

Are sufficient details of methods and materials provided to allow replication by others?

Partly

Is the rationale for creating the dataset(s) clearly described?

Yes

Are the datasets clearly presented in a useable and accessible format?

Yes

Are the protocols appropriate and is the work technically sound?

Yes

Reviewer Expertise:

Molecular biology, molecular ecology and genetics, taxonomy, DNA barcoding

I confirm that I have read this submission and believe that I have an appropriate level of expertise to confirm that it is of an acceptable scientific standard.
